# Association of *IL-4* gene *VNTR* variant with deep venous thrombosis in Behçet’s disease and its effect on ocular involvement

**Published:** 2013-03-21

**Authors:** Ahmet Inanir, Sengul Tural, Serbulent Yigit, Goknur Kalkan, Gunseli Sefika Pancar, Helin Deniz Demir, Omer Ates

**Affiliations:** 1 Gaziosmanpaşa University, Faculty of Medicine, Department of Physical Medicine and Rehabilitation, Tokat, Turkey; 2 Ondokuz Mayis University, Faculty of Medicine, Department of Blood Center, Samsun, Turkey; 3 Gaziosmanpaşa University, Faculty of Medicine, Department of Medical Biology, Tokat, Turkey; 4 Gazi Osmanpaşa University, Faculty of Medicine, Department of Dermatology, Tokat, Turkey; 5 Tokat Public Hospital, Department of Dermatology, Tokat, Turkey; 6 Tokat Gazi Osmanpaşa University, Faculy of Medicine, Department of Ophthalmology, Tokat, Turkey

## Abstract

**Purpose:**

Behçet’s disease (BD) is a systemic vasculitis characterized by inflammatory lesions of the urogenital mucosa, eyes, skin, central nervous system, and joints. Vein thrombosis constitutes the most frequent vascular manifestation of the disease, and may cause such ocular vascular thrombotic events as central retinal vein and central retinal artery thrombosis. Thrombosis is a serious problem, and often leads to irreversible vision loss. Previous studies have shown that genetic factors predispose individuals to BD. Several cytokine genes might play crucial roles in host susceptibility to BD and to thrombophilia. Various polymorphic regions of the interleukin-4 (*IL-4*) gene (−1098G and 590T) are associated with BD in the Turkish population. This study was conducted in Turkish patients with BD to determine the frequency of the *IL-4* gene 70 bp variable number of tandem repeats (VNTR) variant, and its association with clinical findings.

**Methods:**

Genomic DNA obtained from 488 individuals (238 patients with Behçet’s disease and 250 healthy controls) was used in the study. Genomic DNA was isolated and genotyped using PCR assay for the *IL-4* gene 70 bp VNTR polymorphism determined by using PCR with the specific primers.

**Results:**

There was statistical significance between the groups regarding *IL-4* genotype distribution (p<0.001, odds ratio: 2.55 [1.629–4.052], 95% confidence interval) and allele frequencies (p<0.0012.381[1.586–3.617], 95% confidence interval). When we examined *IL-4* genotype frequencies according to the clinical characteristics, we observed a statistically significant association between the P_2_P_2_ genotype and deep venous thrombosis (p=0.01). Deep venous thrombosis was also associated with ocular involvement in our study group (p=0.014).

**Conclusions:**

Our findings suggest that the *IL-4* gene 70 bp VNTR polymorphism is associated with susceptibility to development of BD. Deep venous thrombosis is also associated with ocular involvement in BD. The *IL-4* gene could be a genetic biomarker in Behçet’s disease in a Turkish study population.

## Introduction

Behçet’s disease (BD) is a chronic multisystem inflammatory disorder characterized by mucocutaneous, ocular, vascular, and central nervous system manifestations. The common manifestations are recurrent oral and genital ulcers and ocular involvement. Venous or arterial thromboses occur in 7% to 38% of patients [1]. Venous thrombosis is more common than arterial thrombosis, with relative frequencies of 90% and 10%, respectively [2,3]. Although vascular lesions are not included in the major diagnostic criteria of BD, one-quarter to one half of patients are likely to develop this complication [4-6]. Venous thrombosis is a major vascular involvement reported in 7% to 33% of patients with BD [6]. BD has a worldwide distribution but is most common in Japan, the Middle East, and Mediterranean countries. The prevalence of BD in Turkey is particularly high, at 80–420 per 100,000 individuals [7,8]. BD occurs more commonly in men than in women and primarily affects individuals between the second and fourth decades of their life, with a more aggressive course in young male adults. BD is characterized by infiltration of lymphocytes and neutrophils into the affected organs. Cytokines play critical roles in the pathogenesis of BD [9,10]. Several cytokine genes may play crucial roles in host susceptibility to BD, because cytokine production capacity varies among individuals and depends on the cytokine gene polymorphisms [11]. Cytokines are signaling molecules that contribute to inflammatory response and protect the body from pathogens and other environmental factors. Interleukin-4 (IL-4) is a key cytokine that induces the activation and differentiation of B cells and is involved in the development of the T helper-2 subset of lymphocytes. IL-4 has cytotoxic, antitumor effects, inhibits induction of nitric oxide synthase, inhibits release of superoxide by macrophages, and has numerous anti-inflammatory effects [12-14]. IL-4 also plays a role in the function of macrophages, B-cell and T-cell chemotaxis, the formation of endothelial cell adhesion molecules, and hematopoiesis. Based on these findings, we hypothesized that the genotype of IL-4 in patients with BD may be a determining factor in BD pathogenesis.

## Methods

### Study population

The present study included 238 patients with BD and 250 controls, recruited from the Gazi Osmanpaşa University Department of Physical Medicine and Rehabilitation (Tokat, Turkey). The ethics committee of Gazi Osmanpaşa University Medical Faculty approved informed consent in accordance with the study protocol. Patients with BD fulfilled the International Criteria of Behçet’s Disease for classification [15]. All patients signed a written consent form after being informed about the details of the study. A complete clinical evaluation was performed for all patients. The controls were selected by excluding a diagnosis of BD. All the individuals in the control group were healthy. The data collection sheet included information such as age, disease duration, deep venous thrombosis, and several clinical characteristics. Individual features of patients with BD and controls are summarized in Table 1 and Table 2. Genotype determination DNA was extracted from 2 ml venous blood according to the kit procedure (Sigma-Aldrich,Taufkirchen, Germany) and stored at −20 °C. To detect 70 bp VNTR polymorphism in the third intron of the IL-4 gene, PCR assay was used as described by Mout et al. [16]. PCR was performed with a 25 μl reaction mixture containing 50 ng DNA, 20 pM of each primer, 200 mM of deoxynucleotide triphosphate (dNTP), 2.5 mM MgCl_2_, 0.5 U Taq polymerase, 10 mM KCl buffer (Fermentas, Shenzhen, China). Amplification was carried out using primers F5’ AGG CTG AAA GGG GGA AAG C-3’, R5’-CTG TTC ACC TCA ACT GCT CC-3’, with initial denaturation at 95 ºC for 5 min, 30 cycles of denaturation at 94 ºC for 30 s, annealing at 58 ºC for 45 s, extension at 72 ºC for 1 min, and final extension at 72 ºC for 10 min. P_1_P_1_ genotype was homozygous wild type, P_1_P_2_ genotype heterozygous mutant, P_2_P_2_ genotype homozygous mutation type, wild type allele was P_1_ and mutant type allele was P_2_, respectively. The PCR products were visualized on three percent agarose gel stained with ethidium bromide. PCR product was of 183 bp for P_1_ allele and 253 bp for P_2_ allele. In order to validate the accuracy and reproducibility of this method, each PCR reaction included negative and positive controls. The second PCR was performed to confirm samples whose results were not clear. Also, to confirm the accuracy of the genotyping, repeated analysis was performed on randomly selected samples. No discrepancies were found.

**Table 1:** Demographic variables and baseline characteristics of the patients.

**Individual characteristics**	**Mean±SD**	**Min-Max**
Average age of patients	36.36±9.62	20–70
Average age of controls	35.84±11.36	18–65
Disease duration, years	6.87±5.96	1–29

**Table 2:** Clinical findings of BD patients

**Clinical characteristics**	**Number of Patients (%)**
Skin lesions	234(98.3)
Oral ulcers	234(98.3)
Genital ulcers	168(70.6)
Ocular inflammation	180(75.6)
Deep venous thrombosis	71(29.8)
Colchicine use	234(98.3)
Response to colchicine	238(100)
Papulopustule	96(40.3)
Erythema nodosum	63(26.5)

Min-Max: Minimum-maximum values.

### Statistical analysis

Analysis of the data was performed using SPSS 15.0 (SPSS, Chicago, IL) and the OpenEpi Info software package program [17]. Continuous data were given as mean±SD (standard deviation) and (minimum-maximum values). The frequencies of the alleles and genotypes in the patients and the controls were compared with χ^2^ analysis. Odds ratios (ORs) and 95% confidence intervals (CIs) were calculated. A p value smaller than 0.05 (two-tailed) was statistically significant.

## Results

The demographic variables and baseline characteristics of the patients are shown in Table 1 and Table 2. The mean±SD age was 36.36±9.62 in the patient group and 35.84±11.36 in the control group, respectively. There were 114 (47.90%) women and 123 (52.10%) men in the patient group; in the control group, these figures were 155 (62%) and 95 (38%), respectively. Table 3 presents the distribution of IL-4 70 bp VNTR polymorphic genotypes in the patient group and the control group. Our results showed that there was a statistically significant difference between the groups regarding IL-4 genotype distribution (p<0.001) and allele frequencies (p<0.001; Table 3). In the present study, the P_2_ allele was a 2.3-fold risk factor for BD (OR, 95% CI; 2.381 [1.586–3.617]). When we examined the IL-4 genotype frequencies according to the clinical characteristics, we found a statistically significant association between the P_2_P_2_ genotype and deep venous thrombosis according to the genotype frequencies (p=0.01; Table 4). There was also a statistically significant association between the P_2_ allele and deep venous thrombosis according to the allele frequencies (p=0.008; Table 5). We examined the ocular involvement distribution according to deep venous thrombosis and found a statistically significant association between ocular involvement and deep venous thrombosis (p=0.014; Table 6). In addition, we examined the IL-4 gene 70 bp VNTR genotype and allele frequencies according to clinical status and found that cases with ocular involvement (+) and deep venous thrombosis (+) were associated with the P_2_ allele (p<0.01; Table 7). The IL-4 gene P_1_ allele frequency was 16% in the patient group and 7% in the control group. P_2_ allele occurrence was 84% in the patient group and 93% in the control group (Table 3). The frequencies of the P_1_P_1_, P_1_P_2_, and P_2_P_2_ genotypes of the intron 3 VNTR polymorphism in the patient group were 3.4%, 26.1%, and 70.5%, respectively, and 1.2%, 12.8%, and 86.0%, respectively, in the control group. The homozygote P_1_P_1_ genotype frequency of the IL-4 gene was 3.4% in the patient group and 1.2% in the control group. The heterozygote P_1_P_2_ genotype frequency was 26.10% in the patient group and 12.8% in the control group. The homozygote P_2_P_2_ genotype frequency was 70.50% in the patient group and 86% in the control group.

**Table 3:** Distribution of *IL-4* gene 70 bp VNTR polymorphism and allele frequencies between BD patients and controls.

**Genotype**	**Patients** **(n=238; %)**	**Controls** **(n=250; %)**	**χ2**	P value	OR (95%CI)
P_1_P_1_	8 (3.40)	3 (1.2)	17.33	**p<0.001**	
P_1_P_2_	62 (26.10)	32 (12.8)			
P_2_P_2_	168 (70.50)	215 (86)			
P_1_P_1_+ P_1_P_2_: P_2_P_2_	70:168	35:215	17.15	**p<0.001**	2.555 (1.629–4.052)
I P_1_P_2_+ P_2_P_2_: P_1_P_1_	230:8	247:3	2.585	p=0.107	0.349 (0.074–1.295)
**Allele frequency**					
P_1_	78 (16)	38 (7)	17.98	**p<0.001**	2.381 (1.586–3.617)
P_2_	398 (84)	462 (93)			

The results that are statistically significant are typed in bold. (Homozygous wild type (P_1_P_1_) heterozygous mutant type (P_1_P_2_) homozygous mutation type (P_2_P_2_), Wild type allele (P_1_) mutant type allele (P_2_).

**Table 4:** *IL-4* genotype frequencies according to the clinical characteristics in BD patients (n=238).

**Clinical**		***Il-4 genotypes***			
**Characteristics**		P_1_P_1_	P_1_P_2_	P_2_P_2_	***P* value**
Oral ulcers	yes	9(3.81)	45(19.06)	182(77.11)	p>0.05
	no	-	1(50)	1(50)	
Genital ulcers	yes	4(2.4)	34(20.3)	129(77.2)	p>0.05
	no	5(7.04)	11(15.49)	55(77.46)	
Ocular involvement	yes	4(3.88)	32(31.06)	67(65.04)	p>0.05
	no	4(2.96)	30;(22.22)	101(78.81)	
Deep venous thrombosis	yes	4(9.75)	14(34.14)	23(56.09)	**p=0.01**
	no	4(2.03)	48(24.36)	145(73.60)	
Skin lesions	yes	3(2.88)	31(29.80)	70(67.30)	p>0.05
	no	5(3.73)	31(23.13)	98(73.13)	
Response to colchicine	yes	7(3.44)	37(18.22)	159(78.32)	p>0.05
	no	2(5.71)	9(25.71)	24(68.57)	
Papulopustule	yes	4(4.16)	21(21.87)	71(73.95)	p>0.05
	no	5(3.52)	25(17.60)	112(78.87)	
Erythema.nodusum	yes	9(3.84)	43(18.37)	182(77.77)	p>0.05
	no	-	2(50)	2(50)	

The results that are statistically significant are typed in bold. Wild type allele (P_1_) mutant type allele (P_2_).

**Table 5:** *IL-4* allele frequencies according to the clinical characteristics in BD patients (n=238).

**Clinical**		***Il-4 alleles***		
**Characteristics**		P_1_	P_2_	***P* value**
Oral ulcers	yes	63(13.34)	409(86.65)	p>0.05
	no	1(25)	3(75)	
Genital ulcers	yes	42(12.57)	292(87.42)	p>0.05
	no	21(14.78)	121(85.21)	
Ocular involvement	yes	40(19.41)	166(80.58)	p>0.05
	no	38(14.07)	232(85.92)	
Deep venous thrombosis	yes	22(26.82)	60(73.17)	**p=0.008**
	no	56(14.21)	338(85.78)	
Skin lesions	yes	37(17.78)	171(82.21)	p>0.05
	no	41(15.29)	227(84.70)	
Response to colchicine	yes	51(12.56)	355(87.43)	p>0.05
	no	13(18.57)	57(81.42)	
Papulopustule	yes	29(15.10)	163(84.89)	p>0.05
	no	35(12.32)	249(87.67)	
Erythema.nodusum	yes	61(13.03)	407(86.96)	p>0.05
	no	2 (25)	6(75)	

The results that are statistically significant are typed in bold. Wild type allele (P_1_) mutant type allele (P_2_).

**Table 6:** Ocular involvement distribution according to deep venous thrombosis.

**Clinical status (n,%)**	**Deep venous thrombosis (+)**	**Deep venous thrombosis (-)**	**Total (n,%)**	**χ^2^**	**P value**
Ocular involvement (+)	92 (38.65%)	11 (4.62%)	103 (43.27%)		
Ocular involvement (-)	105 (38.65%)	30 (12.60%)	135 (56.72%)	5.459	**0.014**
Total (n,%)	197 (82.77%)	41 (17.22%)	238 (100%)		

The results that are statistically significant are typed in bold

**Table 7:** Distribution of IL-4 gene 70 bp VNTR genotype and allele frequencies according to clinical status.

Clinical status	Genotype (n,%)			χ2	P value	Allele (n,%)		χ2	P value
	P_2_P_2_	P_2_P_1_	P_1_P_1_			P_2_	P_1_		
Ocular involvement (+) Deep venous thrombosis (+)	6 (2.6)	4 (1.6)	1(0.5)	2.018	p=0.365	16 (3.4)	6 (1.2)	41.34	p<0.001
Ocular involvement (-) Deep venous thrombosis (-)	162 (68)	58 (24.4)	7 (2.9)			382 (80.2)	72 (15.2)		

The results that are statistically significant are typed in bold. Homozygous wild type (P_1_P_1_) heterozygous mutant type (P_1_P_2_) homozygous mutation type (P_2_P_2_), Wild type allele (P_1_) mutant type allele (P_2_).

## Discussion

Behçet’s disease is a multisystem vasculitis that can affect all sizes of blood vessels [18-22]. The disease was first defined by Hulusi Behçet, a Turkish professor of dermatology, in 1937 as a triad of recurrent aphthous stomatitis, genital aphthae, and relapsing uveitis [23]. BD has been reported worldwide but has a distinct geographic distribution with highest prevalences in countries along the ancient Silk Road route [23]. No specific pathological testing or technique is available for diagnosing the disease [23]. Ocular disease is usually bilateral and characteristically occurs within 2 to 3 years of disease onset. Ocular involvement is reported in 30% to 70% of patients with Behcet’s disease. Uveitis involving the anterior and posterior uveal tracts is a significant cause of morbidity. Uveitis, posterior uveitis, and retinal vasculitis may cause visual loss in up to 25% of patients [24]. Although the etiology of BD is not yet known, immune dysregulation is critical factor in the pathogenesis [11]. Genetic factors that predispose individuals to BD play especially important roles in the development of the disease. Familial aggregation studies in patients with BD indicate a strong genetic background and a complex inheritance model [25]. There is a strong association with HLA-B51 and an increased incidence among close family members [26-28]. Venous thrombosis, a clinical finding of BD, is also a common multifactorial disease associated with a major public health burden. Vascular lesions, in particular subcutaneous thrombophlebitis and deep vein thrombosis, may also occur, being detected in 10% to 30% of patients with active disease [4,5]. Vasculitis is the pathological lesion underlying most clinical manifestations of BD, including venous thrombosis. However, thrombophilia may also play an important role in the pathogenesis of the thrombotic manifestations observed in BD [6]. Retinal vein occlusion is associated with increased levels of vascular endothelial growth factor; antivascular endothelial growth factor therapy has been proposed as a promising strategy for retinal vein occlusion [29]. Genetic factors are known to contribute to the susceptibility to venous thrombosis, but how many genes are involved and their contribution to venous thrombosis risk remain obscure [30]. For BD, possible candidate antigens include vascular proteins, because the central histopathological finding in BD is a vasculitis, and environmental factors such as infectious agents, which may cause cross-reactivity to human antigens and result in immune activation [24].

Several previous studies have shown that cytokines play critical roles in the pathogenesis of BD, because cytokines mediate many of the effector and regulatory functions of immune and inflammatory responses [24]. Single nucleotide polymorphisms and a VNTR are the common polymorphisms found in the human genome and cause various disorders [31]. Ollier et al. wrote that variation in cytokine level has been correlated with disease susceptibility and progression [32]. This can be unraveled by investigating cytokine gene polymorphisms to determine whether a genetic basis for cytokine dysregulation is associated with disease. The genetic markers used most often in studies are either microsatellite repeat polymorphisms [33] or single nucleotide polymorphisms (SNPs) [34]. SNPs located in the intronic regions of genes can have no effect on either the level or quality of the protein produced, or, if positioned within an area influencing messenger ribonucleic acid splicing, lead to different splice variants. An *IL-4* gene polymorphism has been reported for its association with several diseases such as Graves disease [1], subacute sclerosing panencephalitis [2], rheumatoid arthritis [3-4], end-stage renal disease [5], idiopathic thrombocytopenic purpura [6], chronic polyarthritis [7], fibromyalgia [8), malaria [9], transitional cell carcinoma of the urinary bladder [10], oral cancer [11], and gastric cancer [12,35]. Several studies have investigated VNTR polymorphisms in different diseases [31,35-39]. Based on these findings, we decided to investigate the effect of the 70 bp VNTR polymorphism on the third intron of the *IL-4* gene in Behçet’s disease.

In this study, the distribution of the *IL-4* gene polymorphic genotypes was analyzed in patients with BD in a Turkish population to assess the possible role of these genotypes in the pathogenesis of BD. The present study indicates that the percentage of the *IL-4* polymorphism allele and the distribution of genotypes differed significantly between the patient group and the control group. When we examined *IL-4* genotype frequencies according to the clinical characteristics, we found a statistically significant association between the P2P2 genotype and deep venous thrombosis. Venous involvement is observed in 25% of patients with BD. Vascular lesions include arterial aneurysms, small-vessel vasculitis, and arterial and venous thrombosis. Venous thrombosis is more common than arterial thrombosis, deep vein thrombosis being the most frequent type of venous thrombosis [40]. The mechanism of the thrombosis in BD is not yet clearly understood. Venous thrombosis and inflammation are two closely related entities [41]. Previous studies have shown that levels of inflammatory substances known as cytokines are raised around the time of a thrombosis [42-44]. In addition, specific polymorphisms in cytokine genes are risk factors for venous thrombosis [42-44]. In this study, the deep venous thrombosis ratio was 29.8%. Men are more severely affected than women. In the present study, the male:female ratio was 1:1.1. The incidence rate of BD is higher in patients’ family members than in the general population [4]. Therefore, genetic analysis is important to elucidate the pathogenic mechanism.

In previous studies, overexpression of proinflammatory cytokines from various cellular sources seemed to be responsible for the enhanced inflammatory reaction in BD, and this may be associated with the genetic susceptibility [40]. Oral et al. investigated *IL-4* and *IL-4Ra* gene polymorphisms, which are different from our polymorphic region, and found that the frequency of *IL-4* −1098 TG and 590 CT genotypes was higher in the patients with BD compared to healthy controls [11]. Analysis of allele frequencies showed that *IL-4* −1098 G and *IL-4* 590 T alleles were more common in patients with BD when compared to healthy controls.

They also reported that the *IL-4RA* gene polymorphism seems to confer pathergy test positivity in patients with BD, whereas none of the *IL-4* gene polymorphisms were associated with clinical findings and specific diagnostic tests for BD [11]. Kurata et al. reported that SNPs rs9261365 and rs2074474 were associated with BD independently of HLA-B51 and - A26 [45]. Akman et al. showed that the tumor necrosis factor-α –1031C allele is associated with susceptibility to BD in the Turkish population [46]. Kim et al. indicated that the interaction of specific rs2275913 in *IL-17A*, *IL-23R*, and rs7574865/rs11889341/rs11685878 in *STAT-4* SNPs modulate susceptibility to intestinal BD in the Korean population, suggesting that the IL-17/23 axis plays a significant role in disease pathogenesis [47]. Recently, genome-wide association studies revealed that variants rs12119179/rs1554286 in *IL-10* and rs1495965 *IL-23R-IL-12RB2* are associated with BD [48,49]. Other studies also identified a strong relationship between the polymorphisms of rs17375018 *IL-23R* and *IL-17* and BD [50,51]. Ozcimen et al. performed a study in Turkish patients with BD to determine the influence of single nucleotide polymorphisms in *IL-1A*, *IL-1B*, *IL-1R*, and *IL-1RA* on disease susceptibility [10]. The authors demonstrated that the *IL-1b* +3962 gene polymorphism seems to be associated with the presence of erythema nodosum in patients with BD. To the best of our knowledge, no reports have been published regarding the role of the *IL-4* 70 bp VNTR polymorphism in BD. Chen et al. reported that the plasma soluble endothelial protein C receptor (sEPCR) level was associated with the polymorphism of *EPCR* gene 6936A/G. The plasma sEPCR level in patients with deep venous thrombosis was higher than that in healthy control subjects [52]. Shahram et al. examined the *IL-2* (−330, +166), *IL-4* (−1098, −590, −33), *IL-10* (−1082, −819, −592), *IL-12* (−1188), *interferon-γ* (5644), transforming growth factor (TGF)-β (codon 10, 25), and *IL-4RA* (+1902) polymorphisms, and reported a significantly increased frequency of *IL-2* (−330) GG genotype (p<0.001), *IL-4* (−33) CC genotype (p<0.001), and *TGF-β* (codon 10) CC genotype (p=0.004). A significant decrease in the frequency of the *IL-4* (−33) TC genotype (p<0.001) was reported in the patient group compared with healthy controls. The genotype CC of *TGF-β* at codon 10 was also significantly overrepresented in the patient group (p=0.004) [53]. Recent studies have suggested that the IL-23/IL-17 axis may be crucial to BD development. A study showed that the expression of IL-23p19 messenger ribonucleic acid, IL-23, IL-17, and interferon-c was markedly elevated in patients with BD with active uveitis [54]. In another study, nuclear factor κB (NF-κB) essential modulator (NEMO), heterozygous (1217A>T, D406V) *NEMO* mutation is a cause of familial occurrence of Behcet’s disease in female patients [55]. Ghioni et al. investigated potential associations between A-13G and G79A polymorphisms of the *protein Z* gene and venous thrombosis and other clinical manifestations in Italian patients with BD. However, no associations were found [56]. Cho et al. demonstrated that the heterogeneous nuclear ribonucleoprotein A2/B1 is a target protein of serum antiendothelial cell immunoglobulin A antibody in patients with BD. Reactivity of serum immunoglobulin A against human recombinant heterogeneous nuclear ribonucleoprotein A2/B1 was detected in 83.3% of patients with BD, whereas it was detected in 0% to 30% of healthy people and disease controls [57]. In a study performed in Turkey, the possible roles of methylenetetrahydrofolate reductase gene C677T, factor V gene G1691A (Leiden), and prothrombin gene G20210A polymorphisms in venous thrombogenesis were evaluated in patients with BD. No association was found among these three thrombogenetic mutations and patients with BD with thrombosis [39]. Several studies have shown that elevated levels of coagulation factors increase the thrombotic risk [58,59]. Increased procoagulant levels might be acquired. Experimental studies in human volunteers injected with low-dose endotoxin provide credence to this possibility, as they showed increases in procoagulant protein levels in parallel with an inflammatory response [60]. Increased levels of inflammatory markers were also found in patients who had had venous thrombotic disease. Inflammation might increase procoagulant protein levels and thus increase the prothrombotic state of the blood [60]. In addition, inflammation may promote tissue factor expression of white blood cells and endothelial cells, thus providing a trigger that may lead to thrombotic disease [60].

In conclusion, our results suggest that possession of the P_1_ allele of the *IL-4* gene 70 bp VNTR polymorphism may constitute a risk for developing BD. Several studies have demonstrated that different genes mutations might play an important role in the etiology of BD. Due to limited research on the *IL-4* gene in BD, the present study makes an important contribution to the literature. Our study demonstrates that polymorphisms in the *IL-4* gene seem to be involved in the susceptibility to BD. Further work is required to confirm these findings in different study groups.
